# Convolutional neural network transformer (CNNT) for fluorescence microscopy image denoising with improved generalization and fast adaptation

**DOI:** 10.1038/s41598-024-68918-2

**Published:** 2024-08-06

**Authors:** Azaan Rehman, Alexander Zhovmer, Ryo Sato, Yoh-suke Mukouyama, Jiji Chen, Alberto Rissone, Rosa Puertollano, Jiamin Liu, Harshad D. Vishwasrao, Hari Shroff, Christian A. Combs, Hui Xue

**Affiliations:** 1 Office of AI Research, National Heart, Lung and Blood Institute (NHLBI), National Institutes of Health (NIH), Bethesda, MD 20892 USA; 2 Center for Biologics Evaluation and Research, U.S. Food and Drug Administration (FDA), Silver Spring, MD 20903 USA; 3 Laboratory of Stem Cell and Neurovascular Research, NHLBI, NIH, Bethesda, MD 20892 USA; 4 Advanced Imaging and Microscopy Resource, NIBIB, NIH, Bethesda, MD 20892 USA; 5 Laboratory of Protein Trafficking and Organelle Biology, NHLBI, NIH, Bethesda, MD 20892 USA; 6 Janelia Research Campus, Howard Hughes Medical Institute (HHMI), Ashburn, VA USA; 7 Light Microscopy Core, National Heart, Lung, and Blood Institute, National Institutes of Health, 9000 Rockville Pike, Bethesda, MD 20892 USA; 8 Health Futures, Microsoft Research, Redmond, Washington 98052 USA

**Keywords:** Imaging, Microscopy

## Abstract

Deep neural networks can improve the quality of fluorescence microscopy images. Previous methods, based on Convolutional Neural Networks (CNNs), require time-consuming training of individual models for each experiment, impairing their applicability and generalization. In this study, we propose a novel imaging-transformer based model, Convolutional Neural Network Transformer (CNNT), that outperforms CNN based networks for image denoising. We train a general CNNT based backbone model from pairwise high-low Signal-to-Noise Ratio (SNR) image volumes, gathered from a single type of fluorescence microscope, an instant Structured Illumination Microscope. Fast adaptation to new microscopes is achieved by fine-tuning the backbone on only 5–10 image volume pairs per new experiment. Results show that the CNNT backbone and fine-tuning scheme significantly reduces training time and improves image quality, outperforming models trained using only CNNs such as 3D-RCAN and Noise2Fast. We show three examples of efficacy of this approach in wide-field, two-photon, and confocal fluorescence microscopy.

## Introduction

The field of microscopy imaging is expanding rapidly with advancements in both software and hardware. These advancements have allowed biologists to image close to the molecular level^1^. Similarly, developments in light-sheet microscopy have allowed for faster and more gentle imaging than ever before^2^. These transformative breakthroughs, alongside constant improvements in fluorescent dyes, keep enhancing the quality of microscopy imaging. However, despite these remarkable advances, the underlying fluorescence signal still constrains the spatial and temporal resolution. Additionally, low illumination intensity is often preferred to avoid photobleaching or cellular phototoxicity^3^, especially for live samples where rapid imaging at low light levels is required to avoid motion artifacts and maintain sample health.

**Figure 1:**
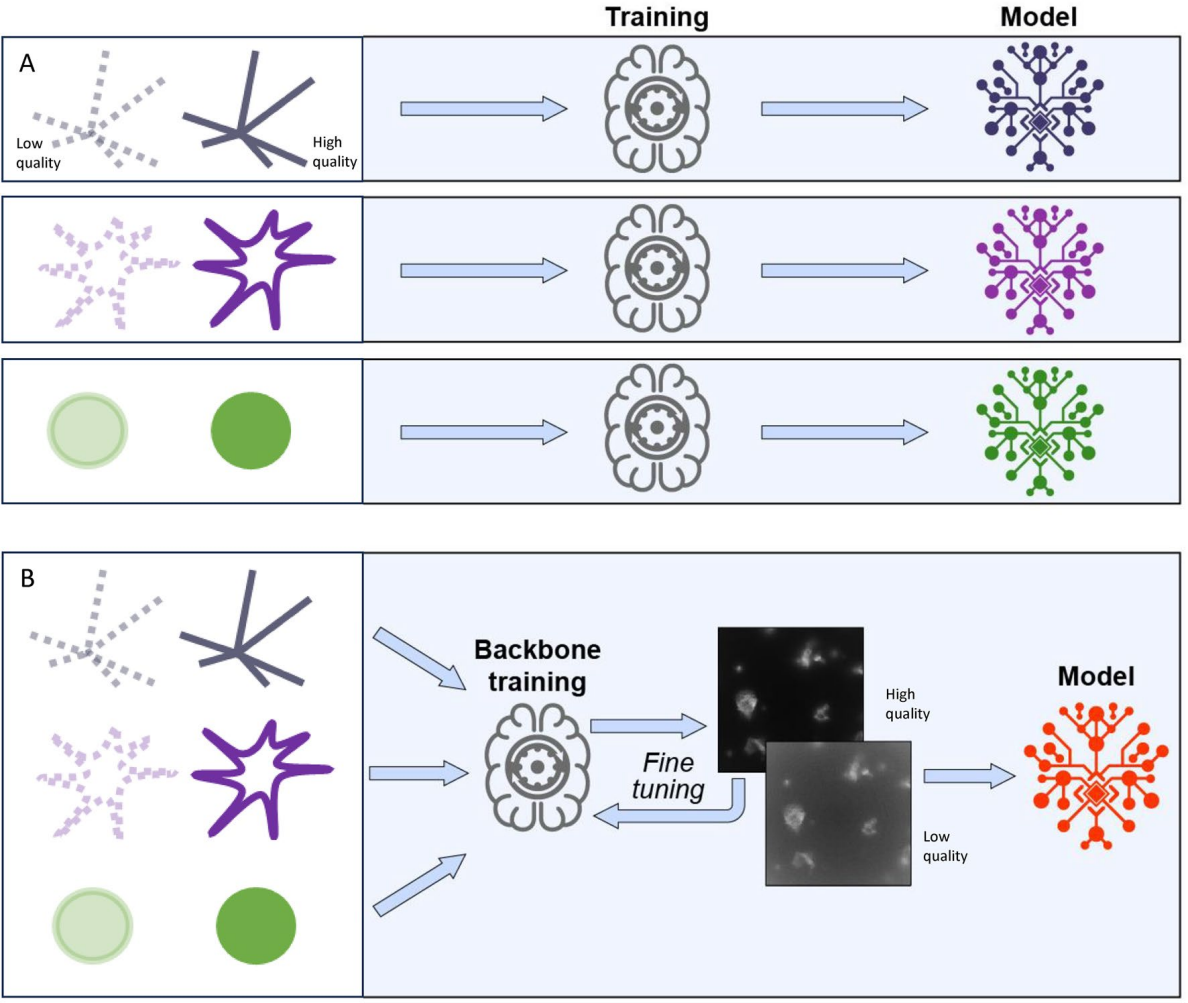
Backbone and finetuning to train the light microscopy image enhancement model. (**A**) Previous methods generally train a separate model for every sample or microscopy type. Such training from scratch methods are effective but need many samples and an extended training time to reach optimal performance. Furthermore, since every training set is independent, the model cannot use the other samples or microscopy type to help the current imaging experiment. (**B**) Here we first train a backbone model from large, diverse, and previously curated data. The trained backbone model is then fine-tuned for every new experiment, using a much smaller amount of new data. Given an effective backbone model architecture, this method will be much faster in training, and allows reusing information acquired in previous experiments. Inspired by the success of transformer model in language pre-training, we propose a novel imaging transformer architecture, CNNT, to serve as the effective backbone.

Deep learning algorithms are currently employed for several image processing tasks including segmentation^4^, super-resolution^5^, and contrast generation in label-free imaging^6^. There has been a recent spate of deep learning algorithms that restore or enhance fluorescence imaging where SNR has been degraded, either for higher speed or for lower illumination following the reasons listed above^7–11^. These deep learning algorithms either use pairs of matching high and low SNR images to build a model and restore low SNR images (supervised training) or use the information in the low SNR images themselves (unsupervised training) for the same purpose. In most cases the best results are achieved with supervised training (Fig. 1A). For instance, using a U-net architecture, Content-Aware image Restoration (CARE)^12^ networks enhance image resolution, denoise low SNR data, and minimize resolution anisotropy. The three-dimensional Residual Channel Attention Networks (3D-RCAN)^13^ have provided further gains in image enhancement for volumetric time-lapse imaging (confocal and super-resolution) and when using high resolution expansion microscopy ground truth.

Although supervised CNN models like CARE and 3D-RCAN are effective, they require considerable time and data to train. The main limitation is the lack of fast adaptation, where new models are trained from scratch for every new experiment requiring a dedicated training dataset, considerable computational overhead, and lengthy training times. Furthermore, models trained under a combination of cell types, hardware and imaging protocol are not robustly transferrable to other experiments^13^. This limits adaptability to new experiments for which they have not been trained. In contrast, unsupervised blind zero-shot denoisers including Noise2Void and Noise2Noise and the more recent Noise2Fast, have been shown to denoise images on a per-sample training^14–17^, where a full training is conducted for the low SNR image, without needing a pre-acquired training dataset. Limitations of these methods include the slow inference due to per-sample training and inferior performance compared to supervised training^16^. High-quality data can be degraded with realistic and diverse noise distributions^18^ to mitigate the requirement of acquiring corresponding low quality data.

The transformer architecture is capable of learning long-range signal coherence and is scalable to large datasets^19^. It is the foundation for the very successful large language models, such as ChatGPT^20^. In the imaging domain, transformer-based models show improved adaptation over CNNs. This is due to the attention mechanism, a key component in the transformer, that dynamically computes input data specific coefficients, while CNNs apply fixed parameters to all input data after training^21^. This adaptation ability is further enhanced by the multi-head soft attention in the transformer^19^.

To improve model adaptation for microscopy denoising experiments, we propose a novel transformer architecture which we term Convolutional Neural Network Transformer (CNNT) that can effectively process large microscopy images. Instead of training individual models for each experiment, we train a general backbone model using diverse datasets followed by fine-tuning this generalized backbone for each new experiment (Fig. 1B). This transfer learning strategy^22^ has recently seen use in microscopy image denoising^23–25^. We take it a step further and show that the CNNT fine-tuning offers better performance than CNN models while requiring only a few pairs of image volumes (e.g. 5–10) for each new experiment, and, consequently, greatly reducing the train time (12 folds to less than 10 min using two A100 GPUs). The fine-tuned CNNT approach also works better than a CNNT trained from scratch on the new data as training from scratch fails to converge in such a short time. The pre-trained backbone model can be generalized across different imaging modalities (e.g. from iSIM to two-photon), cell types and imaging protocols. We trained the backbone model on diverse U2OS cell data acquired with iSIM. The trained backbone was then fine-tuned on three tasks: mouse embryonic fibroblast (MEF) cells imaged with wide-field microscopy, zebrafish embryos imaged with a two-photon system, and mouse lung tissue imaged with a confocal microscope. We compared the CNNT performance with 3D-RCAN and Noise2Fast for both image quality and training speed. Further, the mouse lung tissue was imaged in a 5 × 5 tile setup for a large field-of-view. Low SNR images, because of fast acquisition to freeze sample motion, were significantly improved after model inference, leading to a seven-fold boost in imaging speed. Further comparisons to more recent models were conducted using the mouse lung tissue data. These experiments include supervised models of Neuro-Imaging Denoising via Deep Learning (NIDDL)^23^, and Universal Fluorescence Microscopy-based Image Restoration (UniFMIR)^25^, and self-supervised models of DeepCAD Real Time (DeepCAD-RT)^26^, Denoise Voltage Imaging Data (DeepVID)^27^, and Statistically Unbiased Prediction using spatiotemporal information in imaging data (SUPPORT)^28^. Among these models the UniFMIR makes use of transformers and transfer learning. However, UniFMIR uses the Swin transformer^29^ as backbone, that is essentially 2D, while the CNNT is a novel architecture that is able to process 3D images. In addition, UniFMIR pretrains on a few different tasks on top of denoising while our approach focuses solely on denoising. The results, as summarized in Table 1, show that CNNT outperforms all tested contemporaries.

**Table 1:** Additional comparisons on Mouse Lung Tissue. Comparison with more contemporary models. Details of each are provided in the methods section. Metrics used are PSNR (higher the better) and SSIM3D (higher the better). CNNT trained with 20 samples outperforms all the contemporary models. CNNT trained with 5 and 10 samples are only slightly worse.

Name	PSNR	SSIM3D
RCAN3D	30.0046	0.80468
Noise2Fast	29.6509	0.79898
NIDDL	31.1296	0.76314
DeepCAD-RT	29.6327	0.70675
DeepVID	28.9211	0.65396
SUPPORT	30.066	0.69366
UniFMIR	30.4724	0.75677
CNNT with 5 samples	31.1839	0.84641
CNNT with 10 samples	31.1375	0.83484
CNNT with 20 samples	31.9656	0.85432

## Results

**Figure 2:**
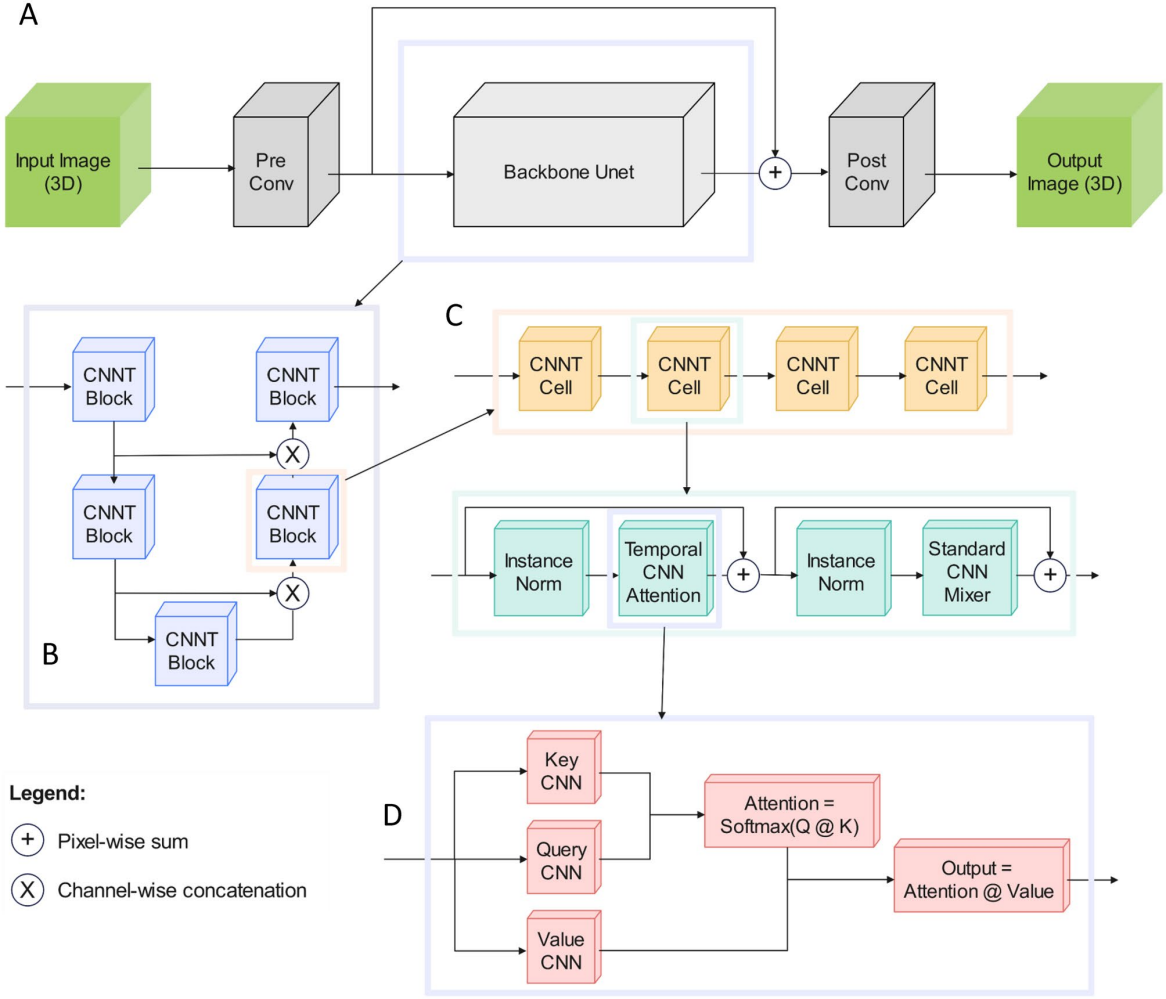
The CNNT U-net architecture. (**A**) The whole model consists of pre and post convolution layers and the backbone. The input tensor has the size of [B, Z, C, H, W] for batch, depth, channel, height, and weight. The C input channel is first uplifted to 32 input channels into the backbone. The post-conv layer will convert the output tensor from the backbone to C channel. There is a long-term skip connection over the backbone. (**B**) The backbone has a Unet structure, consisting of two downsample blocks and two upsample blocks. Every downsample CNNT block will double the number of channels but reduce the spatial size by a factor of two. Every upsample block will reduce the number of channels and expand the spatial size by a factor of two. (**C**) The CNNT block includes only CNNT cells. Every cell contains CNN attention, instance norm and CNN mixer. This design mimics the standard transformer cell design but replaces the linear attention and mixers with CNN attention and CNN mixers, reducing computational cost for high resolution images. (**D**) The CNN attention is the key part of the imaging transformer cell. Unlike the linear layers in the standard transformer, the key, value, and query tensors are computed with convolution layers, which reduces computation cost of processing high-resolution images while also maintaining a good inductive bias. The attention coefficients are computed between query and key and applied to the value tensor to compute attention outputs.

Instead of training new models for every experiment from scratch, a general CNNT (Fig. 2) backbone was trained and then fine-tuned with a few new samples to quickly adapt to a new experiment. In each of the three downstream tasks we finetuned the backbone with 5, 10, or 20 image volume pairs, and compared the CNNT results with 3D-RCAN and Noise2Fast. CNNT was fine-tuned individually for 5, 10, or 20 samples to show indifference in visual performance between the number of samples used to fine-tune. In each fine-tuning case we see a significant boost in SNR compared to the noisy raw image. In addition, CNNT outperforms the current state-of-the-art in both performance metrics, Peak Signal-to-Noise Ratio (PSNR) and three-dimensional Structural Similarity Index (SSIM3D) and training time (fine-tuning time for CNNT, train time for 3D-RCAN, inference time for Noise2Fast). CNNT models show robust adaptation across microscope types, image samples and acquisition conditions, with as few as 5 new volume pairs per experiment.

**Figure 3:**
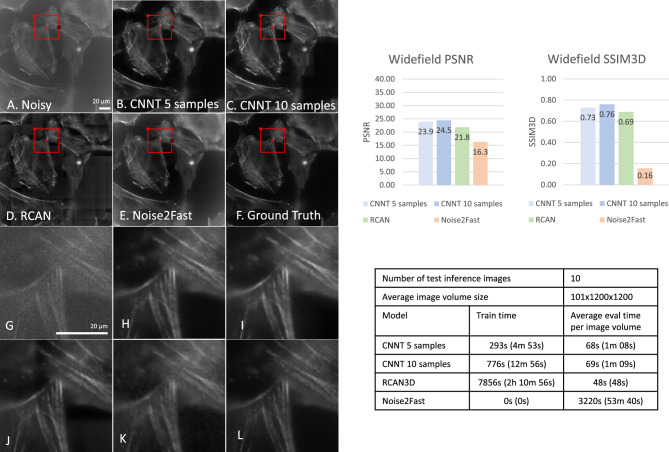
Widefield microscopy experiment, imaging MEF cells. The pre-trained CNNT backbone was finetuned on 5 and 10 widefield image samples individually. The resulting model was compared to 3D-RCAN and Noise2Fast for image quality and computing time. (**A**) The low-quality noisy image as the input to the models. (**B**, **C**) The CNNT results after finetuning for 30 epochs on 5 and 10 samples. The quality improvement is noticeable. (**D**) The 3D-RCAN model trained from scratch for 300 epochs gave good improvement. (**E**) The Noise2Fast result is subpar. (**F**) The high-quality ground-truth for SSIM3D and PSNR computation and for reference. (**G**–**L**) Zoomed in versions of ghted parts in (**A**–**F**), respectively. The CNNT finetuning is much faster than 3D-RCAN training and Noise2Fast and offers better quality measurements.

**Figure 4:**
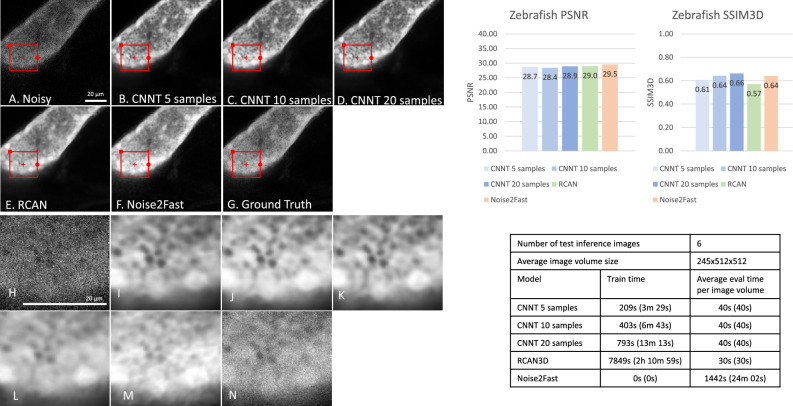
Two-photon microscopy experiment, imaging pancreas of a zebrafish. (**A**) The low-quality image does not provide enough SNR and contrast to delineate the structural features of the pancreas. (**B**, **C**, **D**) The CNNT greatly improved the image quality when using 5, 10, and 20 training samples. The model is robust even for 5 samples, leading to a very fast ~ 3.5 min finetuning time. (**E** and **F**) The 3D-RCAN and Noise2Fast training times are much longer with suboptimal quality recovery. (**G**) The ground-truth in this experiment bears a still lower SNR. (**H**–**N**) Zoomed in versions of highlighted parts in (**A**–**G**), respectively. The CNNT models achieved better quality than the ground-truth images, which could be the result of pre-training.

The fine-tuning for CNNT took approximately 10 min for 10 samples, as seen in Figs. 3, 4, and 6. The inference time is 1 min on large images of size 100 × 1200 × 1200. RCAN has a slower train time of 2 h using the shared codebase and configuration of the large model provided in the shared codebase. Noise2Fast has no train time. Instead, it learns the noise for every data point, leading to very long inference time, upwards of 1 h for large images of size 100 × 1200× 1200.

### Widefield microscopy

Non-muscle myosin 2A-GFP (green fluorescent protein) was imaged in live MEF cells and mouse T cells using widefield microscopy. For this modality, 20 images were collected from which 10 were used for testing. 5 or 10 samples were individually used to fine-tune the CNNT. RCAN was trained on 10 samples as well.

The CNNT restored image quality and uniformly removed noise throughout the field-of-view (MEF cells, Fig. 3). While RCAN also restored image quality, the noise reduction was not uniform (higher noise residue near edges). Noise2Fast did not produce sharp and clean images for the tested dataset. The CNNT shows higher peak signal-to-noise ratio (PSNR) and 3D structural similarity index (SSIM3D) over RCAN and Noise2Fast. Moreover, the fine-tuning time was ~ 13 min for CNNT with 10 samples, while the RCAN training time is over 2 h. Supplementary Video 1 shows the images of non-muscle myosin 2A-GFP in MEF cells and T cells before and after CNNT model was finetuned with ten samples and also includes the ground-truth data for the reference.

### Two-photon microscopy

For this experiment we curated 24 training image volumes focusing on zebrafish liver and pancreas. A hold-out 6 volumes were collected as the test set. Out of 24 training images, CNNT was trained on either 5, 10, or 20 samples. RCAN was trained on all 24. The training was performed on data from sedated zebrafish and the resulting model was successfully applied to live images.

Figure 4 shows CNNT outputs clean images of GFP signal in zebrafish pancreatic tissue with rich details, even visually surpassing the ground truth. This may indicate extra quality gain can be achieved with pre-training. For different numbers of fine-tuned samples, CNNT image quality remains consistent, and better than 3D-RCAN and Noise2Fast. We notice CNNT did not provide the highest PSNR (but was close to other models), because of the noise present in ground-truth images. The fine-tuning time is ~ 6 min for 10 samples, much faster than training from scratch. Supplementary Video 2 shows zebra fish pancreas (left and center column) and liver (right column) results, against the ground-truth.

The reduced imaging time enables motion-free time-lapse volumetric recording of live zebra embryos. Supplementary Video 3 contains the liver images (dsRED + hepatocytes) on top and the exocrine pancreas (GFP + acinar cells) around the Langerhans Islet (big black structure) on the bottom. The raw videos (left column) are noisy due to faster acquisition to freeze motion. The model outputs restored fine structures in the data and provided much higher SNR. For example, in the bottom row, the zymogen granules in acinar cells (the black spots inside the positive signal) are better delineated after the model inference.

**Figure 5:**
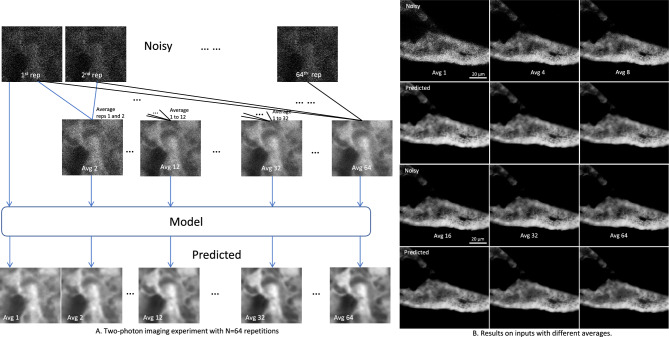
Multi-average tests for the zebrafish imaging with repeated acquisition. (**A**) The imaging was repeated for N = 64 repetitions to image zebrafish liver and pancreas. Averaging the first *n* images creates the image Avg *n*. This gives us a series of images with gradual increase in SNR from Avg 1 to Avg 64. CNNT models were tested for robustness for different levels of input quality with increasing number of averages. The zebrafish liver are shown here. The predicted result for Avg 1 (the lowest quality input) shows residual noise, indicating the model “breaks” at this input SNR. Starting from the Avg 2, mode gives consistently good quality outputs. (**B**) The pancreas results are shown for Avg 1, 4, 8, 16, 32 and 64. In this case, the model was robust against the lower input SNR and recovered finer features.

Time point average experiment was devised to find the breaking point of CNNT U-net. Computing averages for different number of time points creates input images with different levels of SNR. These averaged images are input into CNNT to test its robustness. Figure 5 gives model outputs for averages 1, 4, 8, 16, 32, and 64. We see that CNNT U-net is robust against this wide range of input SNRs. For Avg 1 where the input signal is weak, the model still recovers detailed structures. With improving input SNR of higher averages, the model remained robust, giving consistent high-quality images.

A preliminary study of attention maps was performed using the confocal data. The maps as shown in Supplementary Fig. 1. The maps show interactions between input frames across different resolutions level and cells within a level. While some cells focus on current frame, some focus on neighboring or far away frames. This non-linear combination allows CNNT to produce output frames that are influenced by the entire input image.

### Confocal microscopy

**Figure 6:**
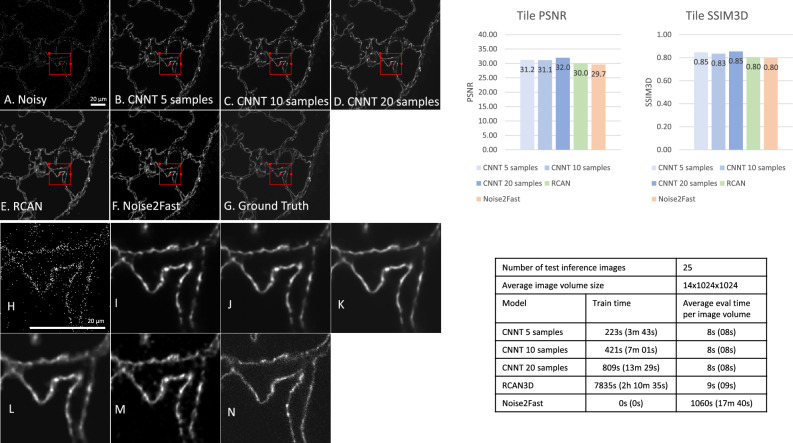
Confocal microscopy, imaging of mouse lung tissue. (**A**) The low-quality image was acquired with very low photon counts. (**B**, **C**, **D**) CNNT finetuning with 5, 10, 20 samples show recovered tissue structures and removal of background random noise. (**E**) The 3D-RCAN model also gave good improvement in quality. (**F**) The Noise2Fast had more signal fluctuation, compared to supervised models. (**G**) The high-quality ground-truth acquisition reveals the tissue anatomical structure. (**H**–**N**) Zoomed in versions of highlighted parts in (**A**–**G**), respectively. Again, the timesaving of CNNT finetuning is prominent, with superior or similar image quality.

In the final experiment we imaged mouse lung tissue with a confocal microscope, imaging 5 different labels (type I alveolar epithelial cells, endothelial cells, immune cells, smooth muscle cells, and nuclei). In this experiment, we tested how much acquisition time could be saved by using the CNNT to denoise very noisy data taken at very high speeds. As shown in Fig. 6, the input raw image has so low SNR that structural information is obscured, while the ground truth shows well connected boundaries. There are a total of 245 image volume pairs made of 49 images from each of the 5 labels (total imaging time is 192 min for ground-truth images and 6 min for noisy images). 25 images, 5 images per label, were separated for testing. From the remaining 220 images CNNT was trained on 5, 10, or 20 randomly selected samples. The 3D-RCAN was trained on all 220 images. Supplementary Video 4 renders five channels to compare images before and after CNNT and the ground-truth scan.

As shown in Fig. 6, the CNNT recovers the image details from very sparsely sampled data, indicating that the backbone training allows the model to learn general structural information on top of denoising. CNNT offers higher PSNR and SSIM3D than RCAN and Noise2Fast, although the RCAN was trained with a lot more samples. Noise2Fast also struggles to produce very clean outputs. The fine-tuning is much faster in training time, ~ 7 min for 10 samples, compared to over 2 h of training from the scratch of 3D-RCAN.

Additional comparisons were made against more recent models using the mouse lung tissue data as well. The results are summarized in Table 1. The train and inference details are given in the methods section. Overall, the models tested were fast in inference (less than 10 s) similar to RCAN3D as most of the models are based on CNNs and the test image size is small as well (14 × 1024 × 1024). However, their performance is slightly worse than CNNT.

## Discussion

In this paper we proposed a novel Convolutional Neural Network Transformer and a backbone training method for light microscopy image denoising. With these contributions, we pushed the boundaries of microscopy image scanning in speed and quality. The backbone model was trained on iSIM datasets and fine-tuned on wide-field, two-photon, and confocal microscopes. Results show that the CNNT architecture and pre-training lead to improved image quality and much less training time, compared to training from scratch. It outperforms the convolution-based models and allows for fast adaptation to new imaging experiments.

The backbone was first trained on a set of iSIM data from seven organelle types and then finetuned on widefield, confocal, and two-photon microscopy experiments. In all three downstream tasks, the CNNT U-net backbone quickly adapted with as few as five new samples. The training method is different from the previously published CNN models that train from scratch. One limitation of training from scratch is that the model cannot use data acquired from other imaging sessions. The backbone training utilizes large amounts of curated data. Based on the scaling law^30^, the performance can be further improved as the amount of training data and model size increases. This might eventually lead to a light microscopy specific foundation model for imaging if a sizable backbone model is pre-trained on a very large and diverse dataset. Resulting models may benefit both imaging and downstream analysis of light microscopy data.

We also studied the robustness of CNNT, via the multiple averaging experiment. Results showed that the fine-tuned CNNT model gave consistently good quality outputs, even for the Avg 1 input with lowest SNR in Fig. 5. In practice, it is plausible to acquire a dataset with multiple repetitions and determine the cutoff with maximal increase in imaging speed by checking the outputs with different input SNRs of different averages. Given the fast fine-tuning time, a new experiment can first determine its optimal imaging setup by quickly training new models from the backbone and then commence the bulk imaging job.

There are some limitations present in this study. First, the dataset used for backbone training was limited to a set of iSIM images. Model performance can be further improved if larger and more diverse datasets are used. Second, a specific model configuration was trained and validated in this study. Future research will work on evaluating model performance against both data and model sizes, to investigate whether the scaling laws of language models apply to light microscopy data or not^30^. Third, the current model was implemented to support tensor in the shape of [B, C, T/Z, H, W] for batch, channel, time or Z-stack, height, and weight. It supports 2D + T or 3D imaging. For the 3D + T imaging, the imaging transformer can be modified to compute attention over 3D images and process tensors in [B, C, T, H, W, Z]. The single 3D volume can be further split into patches for spatial attention. Fourth, the backbone and fine-tuning training was applied to image restoration applications in this study. This training method could be helpful for other microcopy deep learning applications, such as segmentation, tracking, and imaging artifact removal. These are the future research topics.

## Materials and methods

### CNNT: a novel imaging transformer model

CNNs excel at processing image data due to their spatially invariant inductive bias. However, CNNs struggle to capture long range signal correlation along the time or Z axis without significant computational overhead^31^. The standard transformer model performs very well on time series data due to the attention mechanism that can combine all time-points to compute the output. However, it struggles with images due to the heavy computational costs especially when working with 2D + T or 3D images. The standard transformer works with a linear attention mechanism that maps the input to key, query, value (k, q, v) matrices and then computes the attention score. The inputs are mapped to the k, q, v matrices with linear layers. This allows for expressive learning of representations but also incurs a heavy quadratic cost with respect to the input length^32^. We introduce a novel transformer architecture that can work efficiently with 2D + T and 3D images. We use convolution layers instead of linear layers to map the inputs. Not only does this reduce the complexity from a quadratic to linear number of operations, but also introduces the spatially invariant inductive bias of convolutions to the transformer architecture. Convolutions encode each 2D frame of the 3D image into a higher dimension vector space. Transformers take those vectors, flatten them across time, and then use these tensors to compute the attention score on the temporal dimension. This allows richer attention interactions across time and results in better encodings of the complete 3D image. This approach differs from the standard image transformers, Vision Tranformer (ViT)^21^ and Swin^29^, as in these transformer models each individual 2D frame is broken down into patches and the said patches attend to each other via self-attention. However, in CNNT the entire 2D frame is encoded into a vector and then these vectors go through self-attention on the temporal dimension. This novel architecture, coined Convolution Neural Network Transformer (CNNT), allows for several enhancements over CNNs and transformers alone.

As shown in Fig. 2, with the ability to work with flexible dimension sizes as input and output, the CNNT becomes a plug and play module which we use to create complete architectures. As a baseline we create a standalone module of a CNNT cell which consists of an input projection that expands or contracts the channel dimension, followed by a CNNT attention module that enriches the input with convolved attention, followed by a standard CNN mixer that helps in sharing information across feature channels. The modular structure of the CNNT cell becomes the building block for a complete model. We chose U-net as our base architecture as it can effectively combine information from different resolution levels while maintaining computational efficiency. CNNT U-net’s first two levels downscale the image spatially and increase the feature dimension. The last two levels scale up the image spatial dimensions back to the original size and reduce the feature dimension correspondingly. Before each upscale level, we concatenate the input with the output from the corresponding downscale level. Each level of the U-net is made of four CNNT cells stacked on top of each other. We refer to the complete architecture as CNNT U-net.

### Backbone training and finetuning with CNNT U-net

Training was split into two stages of backbone learning and fine-tuning. Instead of training a model for every experiment, we first trained a general backbone with data from several types of experiments pooled together. For fast adaptation to new experiments, a fine-tuning step was performed, requiring only a small dataset and only a few minutes of extra training time. We found that a backbone trained with data from one microscopy type (iSIM^33^ in this study) can be fine-tuned very well on data from other microscopes (e.g. widefield, confocal, two-photon). The backbone training does take longer to complete, but once trained, it can be shared for many new experiments. The fine-tuning takes only a few minutes as the model only needs to adapt to the small new dataset, rather than learn from scratch. In this experiment, CNNT backbone was trained for 300 epochs, while in fine-tuning was further trained for only 30 epochs. In each epoch the model went through a cutout of each image present in the dataset. Since the datasets for fine-tuning are much smaller than the backbone dataset, the train time per epoch for fine-tuning is also considerably less than backbone training (less than a minute compared to 20 min).

We defined a backbone dataset created from imaging a combination of seven different organelle types (Actin, ER, Golgi, Lysosome, Matrix-labelled Mitochondria, Microtubule, and outer membrane-labelled Mitochondria). Three fine-tuning datasets were created from three different samples, acquired from three different microscopes, wide-field, confocal, and two-photon. The seven different organelles used in backbone training were all imaged using iSIM with a total of 154 paired image volumes. None of the fine-tuning data was present in the backbone training. For each downstream task, the CNNT backbone was fine-tuned using either 5, 10, or 20 samples to show the indifference in visual performance between number of samples used to fine-tune.

The model was trained on ssim loss in both the backbone and finetuning. At each iteration, a random cutout was created from the raw images of size 8 × 128 × 128 or 8 × 160 × 160 and used to train the model. Since CNNT does not restrict input shapes it can train with multiple image sizes. Only 2D convolutions with kernel size of 3 × 3 were used throughout the model. The learning rate for the backbone was 1e−4 and for finetune was 2.5e−5. The optimizer used was adamw with beta1 and beta2 values of 0.90 and 0.95 respectively. “Reduce on Plateau” Learning rate scheduler was used with the decay rate of 0.8.

### CNN models for comparison

We compare our results with 3D-RCAN, a state-of-the-art supervised machine learning model, and Noise2Fast, one of the best self-supervised machine learning models. 3D-RCAN was trained from scratch following the directions included in the original publication^13^. Noise2Fast was also run following directions in its original publication. It learns noise prediction for each sample it sees, so no training was required.

### 3D-RCAN

3D-RCAN was trained with the code available online (https://github.com/AiviaCommunity/3D-RCAN). Following the recommendations in its paper, all data available for each downstream task was used to train 3D-RCAN models. The evaluation was done on the same test images as the CNNT. The training and evaluation wall-clock time duration was recorded.

### Noise2Fast

Noise2Fast implementation was downloaded from its official repository (https://github.com/jason-lequyer/Noise2Fast.git). Noise2Fast does not require paired samples; instead, a model is trained from scratch for every noisy image it sees. The published training parameters were used in all experiments.

### Downstream finetuning tasks

### Wide-field imaging of MEF cells

Non-muscle myosin 2A-GFP expressing mouse embryonic fibroblasts (MEF) were maintained in DMEM media (Gibco, Cat# 11965-092), containing 5% FBS (Gibco, Cat# 16000-044). Primary mouse non-muscle myosin 2A-GFP T cells were isolated with EasySep™ Mouse T Cell Isolation Kit, following manufacturer’s instructions (Stemcell, Cat# 19851). Mouse T cells were maintained in RPMI 1640 media (Gibco, Cat# 11875093), supplemented with 10% fetal bovine serum (Gibco, Cat#16000-044) and interleukin-2 (Stemcell, Cat# 78081.1). Isolation of mouse T cells was from 2A-GFP mice was reviewed and approved under ASP# 2020-17 by FDA WO AP IACUC.

For live imaging of non-muscle myosin 2A-GFP in MEF cells and T cells we used widefield Leica DMi8 microscope equipped with LED5 light source (479 nm), GFP filter cube (Ex:470/40, DC: 495, EM:525/50), 100x/1.4 NA oil immersion objective lens, adaptive focus control, and Okolab stage top incubator with CO_2_, temperature, and humidity control. The low and high SNR image pairs of cells were acquired as 25 and 100 ms exposure. The high SNR data was acquired with 20-folds laser power than the low SNR images.

### Two-photon imaging of zebrafish embryos

All zebrafish experiments were performed in compliance with the National Institutes of Health guidelines for animal handling and research using an Animal Care and Use Committee (ACUC) approved protocol H-0252(R5). Zebrafish were raised and maintained at the temperature of 28.5C. Zebrafish handling, breeding, and staging were performed as previously described^34,35^. To prevent pigmentation, the embryos used for confocal analysis were cultured in fish water containing 0.003% 1-phenyl-2-thiouera (PTU, Sigma-Aldrich, P7629) from 24 hpf. The following strain was used: Tg(ins:dsRed)^m1081^;Tg(fabp10:dsRed;ela3l:GFP)^gz12^; Tg(ptf1a:EGFP)^jh1^ transgenic line^36^. Originally, the line was obtained crossing the Tg(ins:dsRed)^m1081^;Tg(fabp10:dsRed;ela3l:GFP)^gz12^ line, also known as 2-Color Liver Insulin acinar Pancreas (2CLIP) with the Tg(ptf1a:EGFP)^jh1^ line.

Two-photon imaging of zebrafish embryos was performed on Tg(ins:dsRed)^m1081^;Tg(fabp10:dsRed;ela3l:GFP)^gz12^; Tg(ptf1a:EGFP)^jh1^ transgenic line at room temperature using a LEICA SP8 confocal microscope and a 25x (0.95 NA) water dipping lens (Leica HC FLUOTAR L VISIR) with a dual beam Insight (Ti:sapphire) laser (Newport/Spectra-Physics, Irvine, CA). The fish express dsRED fluorescent protein in the islets of Langerhans in the endocrine pancreas, driven by the insulin (*ins*) promoter and in the liver hepatocytes, driven by the fatty acid binding protein 10a (*fabp10a* gene). Heterozygous parents were crossed and then the collected embryos were selected at 3 days post fertilization (dpf) using a fluorescent SteREO Discovery.V12 stereomicroscope (Zeiss). At 5 dpf, zebrafish embryos were anesthetized using a buffered tricaine methane sulfonate (MS-222, Sigma-Aldrich, E10521) solution in 0.003% PTU solution in E3 medium. The anesthetized embryos were then included in a 1% solution of low melting agarose dissolved in 0.003% PTU solution in E3 medium on a glass coverslip (Warner Instruments, CS-40R15) and carefully oriented in a lateral position. During imaging embryos were kept in a solution of MS-222 and 0.003% PTU in E3 medium. Pairs of low-noise “ground-truth” and fast “noisy” image stacks of DsRed and GFP were acquired at a scan rate of 8000 Hz using a resonant scanner with a format of 512 × 512 pixels, 0.2 × 0.2 micron pixel sizes, and excitation at 1045 nm (DsRed) and 920 nm (GFP), with emission bandwidths of 650–700 nm (DsRed) and 500–552 nm (GFP), and an interslice distance of 0.5 microns. A line average of 8 was used for the ground truth images whereas no line averages were used for the noisy image stacks resulting in a decreased time of imaging of more than sixfold. All training data were acquired on fixed zebrafish to avoid motion artifacts during the acquisition of ground truth data. The model was then used to restore images taken using the same fast imaging settings on live zebrafish.

We also devised a new experiment to find the breaking point of CNNT. The confocal microscope allows us to image repeatedly and average them afterwards to improve quality. We exploit this to create a series of images with gradually increasing SNR by taking 64 repetitions of the same field-of-view and then averaging the first *n* time points to get the *n*th image, as illustrated in Fig. 5A. We selected time averages 1–63 as the noisy images and the average n = 64 as the ground truth (the best possible quality). We test the fine-tuned model on all time point averages from 1 to 64 to evaluate model behaviors for inputs with different SNR levels. This experimental method can evaluate model robustness against different input quality and reveal the model “breakdown-point”.

We further acquired time-lapse data for zebrafish liver and pancreas at the temporal solution of 75 ms per z step. The z-stack acquisition time is 1.7 s to 3.2 s, depending on the number of z steps. To avoid photobleaching and the motion artifact, acquisition with gentle illumination leads to lower quality data, which was later enhanced by the model. This is to demonstrate the use case for live animal imaging.

### Confocal imaging of mouse lung tissue

Lungs isolated from C57BL/6 mice were inflated with 4% paraformaldehyde/PBS and immersed in 4% paraformaldehyde/PBS at 4 °C overnight. After fixation, the lungs were immersed in 30% sucrose/PBS at 4 °C overnight and then embedded in OCT compound. Cryosectioning of the lung tissues was performed at a thickness of 50 µm and mounted on Superfrost Plus Gold microscope slides (Fisher). Immunostaining was performed with the following primary antibodies: rat anti-CD45 antibody (1:500, eBioscience, 14-0451-85), mouse anti-αSMA-Cy3 (1:500, Millipore Sigma, C6198), armenian hamster anti-PECAM-1/CD31 antibody (1:300, Chemicon, MAB1398z), and syrian hamster anti-Podoplanin-APC (1:50, Biolegend, 127410). For immunofluorescent detection, donkey anti-rat IgG-Alexa 488 (1:250, Invitrogen, A21208) and goat anti-armenian hamster IgG-Cy3 (1:250, Jackson ImmunoResearch, 127-165-160) secondary antibodies were used. Nuclei were visualized with Hoechst 33342 (1:500, Biotium, 40046). Mouse lung tissue was imaged using a Leica SP8 confocal microscope in the resonant scanning mode (8000 Hz) using a 63x/1.4 NA oil objective (Leica HC PL APO CS2) and the LASx software (version 3.5.7). 5 × 5 tiled, five-color, z-stack images were acquired with 144 nm pixels in the XY dimension with a format of 1024 × 1024, an interslice distance of 2 µm, with a pinhole set to 1 A.U. Excitation and emission ranges for Hoechst, Alexa Fluor 488, CY3, PECAM, and APC were 405 with 415–458, 500 with 509–542, 550 with 559–587, 594 with 606–635, and 650 with 660–745 nm respectively. Line-average varied with “ground truth” images collected with a line average of 32 while faster “noisy” test images were collected with a line average set to be 4. The ground-truth images were acquired in ~ 3 h, but noisy data was acquired in 6 min.

### Animal and cell lines

MEF cells were isolated and immortalized previously^37^ with all mouse procedures performed with approval from the National Heart, Lung, and Blood Institute Animal Care and Use Committee and Duke University Medical School Institutional Animal Care and Use Committee. Isolation of mouse T cells was from 2A-GFP mice was reviewed and approved under ASP# 2020-17 by FDA WO AP IACUC. Zebrafish and mouse tissue harvesting for imaging were approved by and performed in compliance with the National Institutes of Health guidelines for animal handling and research using an Animal Care and Use Committee (ACUC) approved protocol (H-0252(R5) and H-0154(R4)) respectively. Animal experiments were performed according to ARRIVE guidelines.

### Additional models for comparison using mouse lung tissue

Additional comparisons were performed with more contemporary studies. The models were either trained from scratch, finetuned, or downloaded from the publications. Individual model details are present below. The comparisons were performed on the Mouse Lung Tissue dataset with 220 images for training and 25 for testing. The other experiments had too few training samples which caused the models to not converge and output gibberish results.

### NIDDL

NIDDL^23^ was trained using the code provided with the paper: https://github.com/shiveshc/NIDDL. Supervised approach that provided pretrained backbones. The backbone model that produced the best results was then finetuned on all of our Mouse Lung Tissue data for 10 epochs and then tested on the test set.

### DeepCAD-RT

DeepCAD-RT^26^ was advertised as an out-of-the-box solution, ready to be used: https://github.com/cabooster/DeepCAD-RT/. A self-supervised approach that provides several pretrained models. All provided models were tested and the best score was recorded.

### DeepVID

DeepVID^27^ code was taken from the published repository: http://github.com/bu-cisl/DeepVID. A self-supervised approach that learns from each set of data separately. This model was trained from scratch on all training data from 200 epochs and then tested on the test set.

### SUPPORT

The published repository was used for SUPPORT^28^: https://github.com/NICALab/SUPPORT. A self-supervised approach that is built on top of the previous research. The model was trained from scratch on all the data present for 100 epochs and tested on the test set.

### UniFMIR

The publicly available code was used for UniFMIR^25^: https://github.com/cxm12/UNiFMIR. A pre-trained model was provided. This model was then finetuned on denoising the Mouse Lung Tissue train set for 30 epochs and then tested on the test set. A supervised approach that trains a backbone on several tasks and then finetunes on the desired one.

### Hardware details

All the models were trained and evaluated on a server running Ubuntu 22.04, with 2 × 80 GB Nvidia A100 GPUs, and 2 × AMD EPYC 7473X 24-Core Processor with 1 TB RAM. Distributed data parallel training was performed in all experiments to use both GPUs simultaneously wherever possible.

### Supplementary Information

Supplementary Figure S1.

Supplementary Video 1.

Supplementary Video 2.

Supplementary Video 3.

Supplementary Video 4.

Supplementary Legends.

## Data Availability

Training and test datasets for widefield, two-photon and confocal experiments are published on the NIH data repo (https://figshare.com/s/7dffc5aca06b52382a04). The 3D-RCAN iSIM datasets were shared with its original publication (https://zenodo.org/records/4624364#.YF4lBa9Kgal). Only “Denoising.zip” was used to the train the backbone model.

## Code availability

The CNNT model and training code used in this study are available at https://github.com/AzR919/CNNT_Microscopy.git. An installation guide, data and instructions for use are also available from the same webpage.
