# Supplementary material for: Convolutional neural network transformer (CNNT) for fluorescence microscopy image denoising with improved generalization and fast adaptation
Source: Sci Rep. 2024 Aug 6;14:18184. doi: 10.1038/s41598-024-68918-2 (PMC11303381; doi:10.1038/s41598-024-68918-2)
Supplement: Supplementary file 1 — Supplementary Figure S1. [file 41598_2024_68918_MOESM1_ESM.pdf]

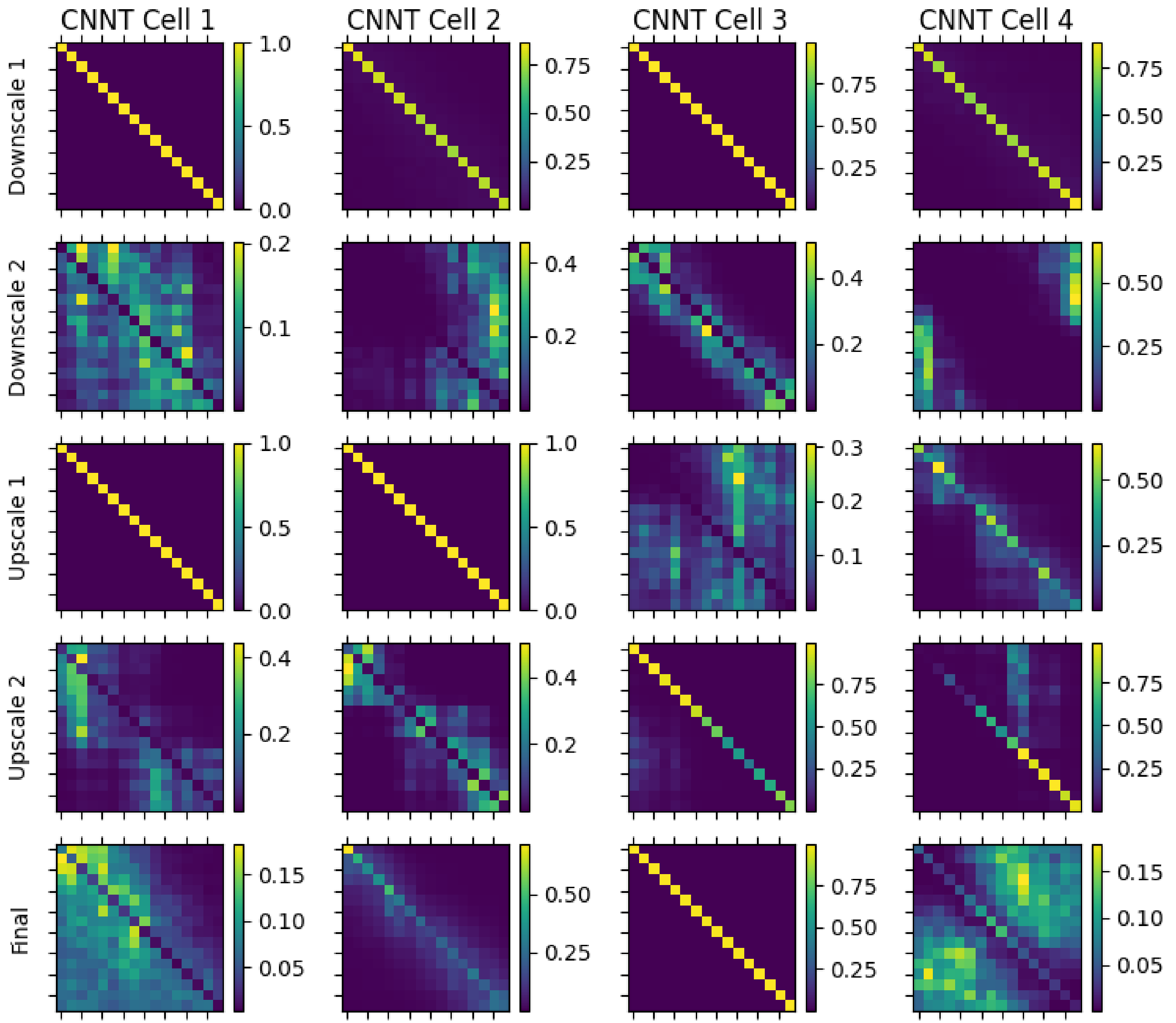


**Supp. Fig. 1 | Attention maps computed by first head of each layer at runtime.** Computed attention maps at runtime throughout the CNNT U-net, five levels of U-net with each level containing four CNNT Cells. Each cell has eight heads, but for ease of view we select to show only the first one. The sample input image had dimensions 16x160x160, so the attention maps have dimensions 16x16, as the attention is computed across time. We see different levels and different heads assume different roles. In the first level each frame focuses solely on itself with heavy weight on the diagonal. In the second level each frame focuses on surrounding or far away frames, specifically avoiding itself. The remaining levels avoid categorization but the cells within them still show noticeable structure. Some focus on same frame, and some avoid current frame and focus on nearby or far away frames. Overall, we see a myriad of interactions that allow CNNT to denoise using the information present throughout the 3D image.
