# Supplementary material for: Convolutional neural network transformer (CNNT) for fluorescence microscopy image denoising with improved generalization and fast adaptation
Source: Sci Rep. 2024 Aug 6;14:18184. doi: 10.1038/s41598-024-68918-2 (PMC11303381; doi:10.1038/s41598-024-68918-2)
Supplement: Supplementary file 6 — Supplementary Legends. [file 41598_2024_68918_MOESM6_ESM.docx]

**Supplementary Video Legends**

**Supp. Video 1 | Widefield microscopy experiment, imaging MEF cells.** The pre-trained CNNT backbone was fine-tuned on 10 widefield image volume pairs. The resulting model was applied to noisy image volumes. The first row shows the noisy raw images, followed by model output in the second row, and then the corresponding ground truth in the third row. The first and third columns show 2A-GFP in MEF cells. The second column shows T cells. The CNNT uniformly removes noise throughout the field-of-view, even when the noise is denser near the edges in the raw images. Scale bar: 20 μm.

**Supp. Video 2 | Two-photon microscopy experiment, imaging of a zebrafish.** The pre-trained CNNT backbone was fine-tuned on 10 two-photon zebrafish image volume pairs. The resulting model was applied to noisy image volumes. The first row shows the noisy raw images, followed by model output in the second row, and then the corresponding ground truth in the third row. The first two columns are zebrafish pancreas and the third column is the liver. The model is able to recover fine details that are hardly visible in the raw images due to fast acquisition. The results are comparable to the ground truth images that had a much longer scan time. Scale bar: 20 μm.

**Supp. Video 3 | Two-photon microscopy experiment, imaging of a live zebrafish.** The pre-trained CNNT backbone was fine-tuned on 10 two-photon zebrafish image volume pairs. The zebrafish was sedated to prevent motion artifacts. The resulting model was applied to noisy image volumes gathered from a live zebrafish. The first column shows the noisy raw images with live motion and low SNR due to fast acquisition. The second column shows the model output on the raw images. The first row contains the liver images (dsRED+ hepatocytes) and the second row contains the exocrine pancreas (GFP+ acinar cells) around the Langerhans Islet (big black structure). The significant boost in SNR means that CNNT allows for quick image acquisition of live samples that can then be denoised to achieve high quality results. Scale bar: 20 μm.

**Supp. Video 4 | Confocal microscopy, imaging of mouse lung tissue.** The pre-trained CNNT backbone was fine-tuned on 25 samples of mouse lung tissue consisting of 5 samples from 5 different labels (type I alveolar epithelial cells, endothelial cells, immune cells, smooth muscle cells, and nuclei). The resulting model was applied to a much bigger 5x5 tile experiment. The first column shows the noisy data acquired in only 6 minutes. The second column shows the output of CNNT. The third column shows the corresponding ground truth that had an acquisition time of 192 minutes.
